# 1033. Management of Disseminated Nocardiosis in an Immunocompromised Patient Using Multi-Drug Therapy

**DOI:** 10.1093/ofid/ofac492.874

**Published:** 2022-12-15

**Authors:** Abrar Khan, Talha Perwez, Junaid Farooq, Ramiro Gutierrez, Nabil Zeineddine, Rahul Mahapatra

**Affiliations:** SUNY Upstate University, Syracuse, New York; SUNY Upstate Medical University, Syracuse, New York; SUNY Upstate Medical University, Syracuse, New York; SUNY Upstate Medical University, Syracuse, New York; SUNY Upstate Medical University, Syracuse, New York; SUNY Upstate University, Syracuse, New York

## Abstract

**Background:**

Nocardiosis may cause fatal infections in immunocompromised and immunocompetent patients. While there are many different species, Nocardia farcinica, has been a clinically important cause but less frequently identified species. We present a case of an immunocompromised patient successfully treated with a multi-drug regimen for disseminated nocardiosis due to Nocardia farcinica.

**Figure:**
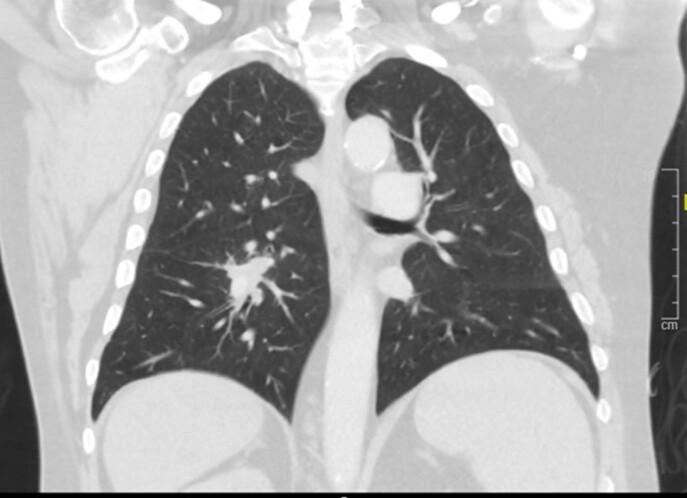

**Figure:**
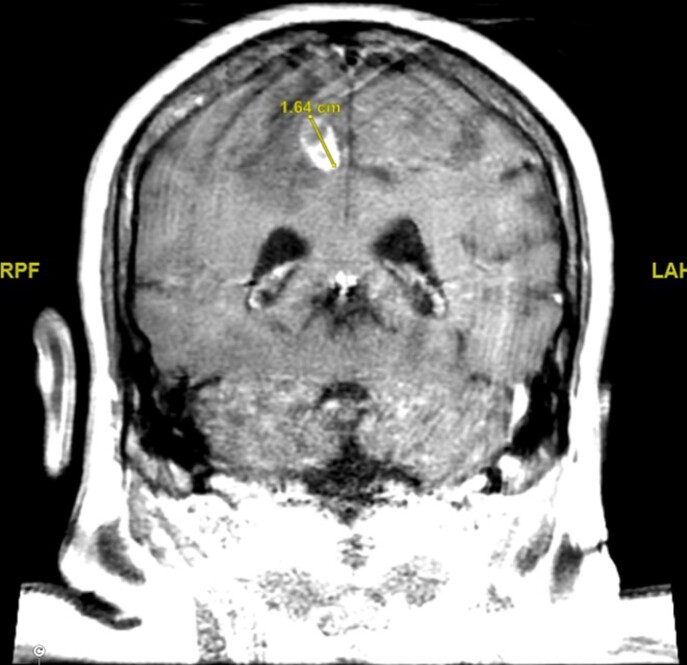

**Methods:**

The patient is a 65-year-old male with a history of Autoimmune Hemolytic Anemia (AIHA) who presented with fevers, chills, symptomatic anemia, and new left lower extremity weakness associated with spasms over several days prior to arrival.

**Figure:**
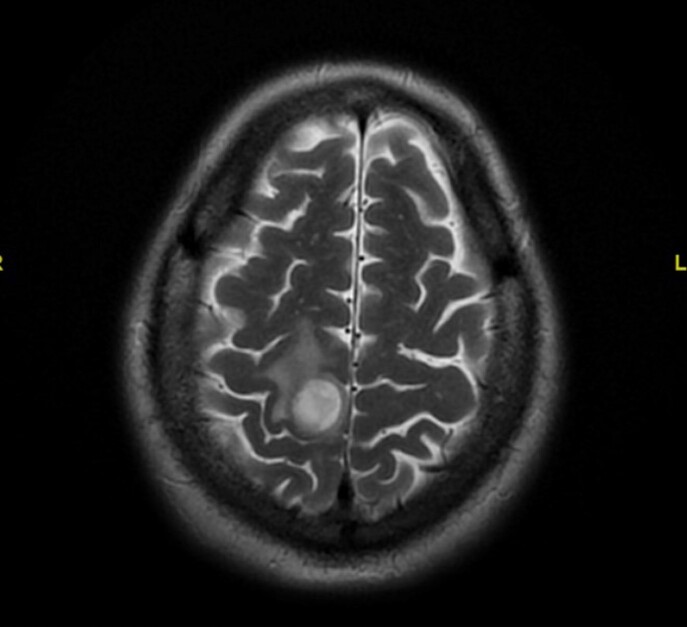

**Results:**

6 months prior, he had received treatment for AIHA with rituximab, cyclophosphamide, and high dose corticosteroids. Head CT revealed a 1.5cm right posterior frontal lobe mass with surrounding edema. CT thorax revealed innumerable spiculated pulmonary nodules and masses bilaterally. Lung biopsy as well as blood culture were positive for Nocardia farcinica, specimen was sent to reference laboratory for susceptibility testing. He was initially treated with imipenem and IV Bactrim. Subsequently, clinical exam findings were unchanged and repeat MRI brain showed enlarging frontal mass. Linezolid and IV amikacin were added to the regimen based on susceptibility results. Patient was discharged home but returned to hospital shortly after discharge with severe hemolytic anemia, suspected to be due to Bactrim administration. The patient was later discharged home on Imipenem, IV amikacin, oral Linezolid, and oral Moxifloxacin in stable condition without surgical intervention and with improved left lower extremity weakness.

**Conclusion:**

Disseminated Nocardiosis is known to cause fatal pulmonary and CNS infections in immunocompromised patients and requires aggressive therapy usually with multiple antibiotics. Nocardia farcinica infections are particularly clinically complex given the organism’s multiple inherent resistance mechanisms, interpretation of in vitro susceptibility testing, and possible adverse effect profile of treatment regimen.

**Disclosures:**

**All Authors**: No reported disclosures.

